# Pushing boundaries in cardiac surgery: minimally invasive mitral valve repair combined with tricuspid valve repair and/or other concomitant procedures

**DOI:** 10.3389/fcvm.2024.1407591

**Published:** 2024-08-09

**Authors:** Marie-Elisabeth Stelzmueller, Robert Zilberszac, Raphael Rosenhek, Doris Hutschala, Sabine Kappel, Andrea Lassnig, Guenther Laufer, Daniel Zimpfer, Wilfried Wisser

**Affiliations:** ^1^ University Clinic of Cardiac Surgery, Medical University Vienna, Vienna, Austria; ^2^ Department of Cardiology, Medical University Vienna, Vienna, Austria; ^3^ Department of Cardiothoracic Anaesthesia and Intensive Care, Medical University Vienna, Vienna, Austria; ^4^ Department of Cardiac Surgery, Medical University Graz, Graz, Austria

**Keywords:** mitral valve repair, tricuspid valve repair, biatrial maze, totally endoscopic, minimal invasive cardiac surgery, 3D video endoscopic minimal invasive surgery

## Abstract

**Introduction:**

Minimally invasive mitral valve repair/replacement has emerged as a widely accepted surgical approach for managing mitral valve disorders. Continuous technological progress has contributed to the refinement of this procedure, leading to improved safety, decreased surgical trauma, and faster recovery times. Despite these advancements, there remains a scarcity of data concerning minimally invasive complex mitral valve repair surgeries when combined with additional procedures.

**Methods:**

Between November 2008 and December 2022, 153 patients underwent an operation using a minimally invasive technique. All patients underwent mitral valve surgery for severe mitral valve insufficiency/stenosis in combination with at least one additional procedure for tricuspid valve repair (*n* = 52, 34%), patent foramen ovale or atrial septal defect closure (*n* = 34, 22.2%), left atrial appendage occlusion (*n* = 25, 16.3%), or electrophysiological procedure (*n* = 101, 66.0%). Two concomitant procedures were conducted in 98 patients (64.1%), three concomitant procedures in 49 patients (32%), and four concomitant procedures in 6 patients (3.9%).

**Results:**

Surgical success was achieved in 99.3% of the patients (*n* = 152), one patient required a revision of the mitral valve repair on the first postoperative day due to systolic anterior motion phenomenon. Mitral valve repair was performed in 136 patients (88.9%), while 15 patients (9.8%) received a mitral valve replacement as per a preoperative decision due to severe mitral valve stenosis, and two patients (1.3%) underwent other mitral valve procedures. Therapeutic success in treating atrial fibrillation was achieved in 86 patients (85.1%) of the 101 who received an additional maze-procedure. The 30-day mortality rate was 0.7%, with one patient succumbing to respiratory failure. Neurological complications occurred in 7 patients (4.6%). Freedom from reoperation was calculated as 98% at 5-year follow-up and 96.5% at 10-year follow-up.

**Conclusion:**

Minimally invasive mitral valve surgery, even when performed alongside concomitant procedures, stands out as a reproducible and safe technique with outstanding outcomes. It is imperative to advance towards the next frontier in minimally invasive surgery, encouraging experienced surgeons to undertake more complex procedures using minimally invasive approaches. These results help envision extending the boundaries of minimally invasive surgery by performing complex mitral valve procedures and associated interventions entirely through endoscopic means in suitable patients.

## Introduction

Advancements in surgical techniques and technology have led to a significant increase in the adoption of minimally invasive mitral valve repair procedures, offering patients the advantages of reduced surgical trauma and shorter recovery periods with excellent perioperative and longterm results (1–8). Notably, these innovations have been demonstrated to maintain comparable risks and repair rates to traditional open sternotomy procedures (8–13). However, skepticism persists due to concerns about prolonged cardiopulmonary bypass (CBP), operation times, limited exposure, and potential compromises in safety (15, 16). Despite these criticisms, many medical institutions remain resolute in their belief in the benefits of less invasive approaches (1–11, 13, 17).

The literature provides evidence supporting the efficacy of minimally invasive mitral valve repair techniques. One of the most popular approaches is the video-assisted method, which involves a right minithoracotomy (2–4, 7, 14, 16). The introduction of 3D endoscopy has further advanced video-assisted procedures, allowing totally endoscopic mitral valve repair and thus reducing the invasiveness of the procedure. This state-of-the-art technology enables a fully endoscopic approach, even in cases involving complex mitral valve repairs or combined procedures (6, 10, 17).

While minimally invasive mitral valve repair procedures are well documented in the literature, there remain limited data on the promising results of mitral valve repair in combination with maze procedures (8, 16, 18–20). For more complex concomitant procedures, there are even fewer publications presenting outcomes and performance metrics for combined procedures (8, 18, 21).

Additionally, there are concerns that the prolonged duration of the operation, bypass, and aortic cross-clamp time may consequently lead to inferior mitral valve repair rates. Against this background, this study was designed to analyze the feasibility of complex combined minimally invasive mitral valve operations, with a focus on perioperative outcomes, surgical success, conversion rates, survival, and freedom from reoperation.

## Patients, materials, and methods

Between November 2008 and October 2022, a total of 464 consecutively sampled patients underwent minimally invasive surgery, primarily for mitral valve insufficiency. Among them, 311 patients received single mitral valve repair or replacement, while 153 patients underwent additional procedures. These 153 patients had a distribution of 63 female and 90 male patients, with a mean age of 63 ± 12 years.

Detailed demographic data are provided in Table 1.

**Table 1:** Patient demographics.

** **	*N* = 153
Age (years)	63 ± 12
Sex	
Male	90 (58.8%)
Female	63 41.2%)
Mitral valve insufficiency	
Grade II	2 (1.3%)
Grade III	4 (2.6%)
Grade IV	147 (96.1%)
Mitral valve stenosis	6 (3.9%)
Endocarditis	4 (2.6%)
Active	2 (1.3%)
Previous	2 (1.3%)
Tricuspid valve insufficiency	
Grade 0	93 (60.8%)
Grade I	5 (3.3%)
Grade II	0 (0%)
Grade III	18 (11.8%)
Grade IV	37 (34.2%)
Left ventricular function (LVEF)	
Very poor <20%	0 (0%)
Poor 21–30%	2 (1.3%)
Moderate 31–55%	12 (7.8%)
Good >50%	139 (90.8%)
Mean LVEF (mmHg)	60.2 +/− 7
Systolic pulmonary artery pressure (sysPAP)	
Moderate 31–55 mmHg	65 (42.5%)
Severe >55 mmHg	53 (34.6%)
Mean sysPAP (mmHg)	46.5 +/−21
Angina (stable)	3 (2.0%)
NYHA	
NYHA I	19 (12.4%)
NYHA II	67 (43.8%)
NYHA III	64 (41.8%)
NYHA IV	3 (2.0%)
Atrial fibrillation	112 (73.2%)
Previous Cardioversion	19 (12.4%)
Previous pulmonary vein ablation	3 (2.0%)
Previous pacemaker implantation	5 (3.3%)
Previous cardiac surgery	2 (1.3%)
Previous cardiac interventions	1 (0.7%)
Diabetes	2 (1.3%)
IDDM	0 (0%)
NIDDM	2 (1.3%)
Kidney Injury	
No (CC>85 ml/min)	64 (61.8%)
Moderate (CC >50–85 ml/min)	66 (43.1%)
Severe (CC<50 ml/min)	23 (15%)
Creatine Clearance (ml/min) mean	82.5 ± 32.6
Previous Stroke	9 (5.9%)
Peripheral artery disease	1 (0.7%)
Cerebral vascular disease	2 (1.3%)
Previous pulmonary embolism	2 (1.3%)
Pulmonary disease	14 (9.2%)
EuroScorelog	4.5 ± 4.7
EuroScoreII	3.2 ± 3.2
Smoker	32 (20.9%)

**Figure 1:**
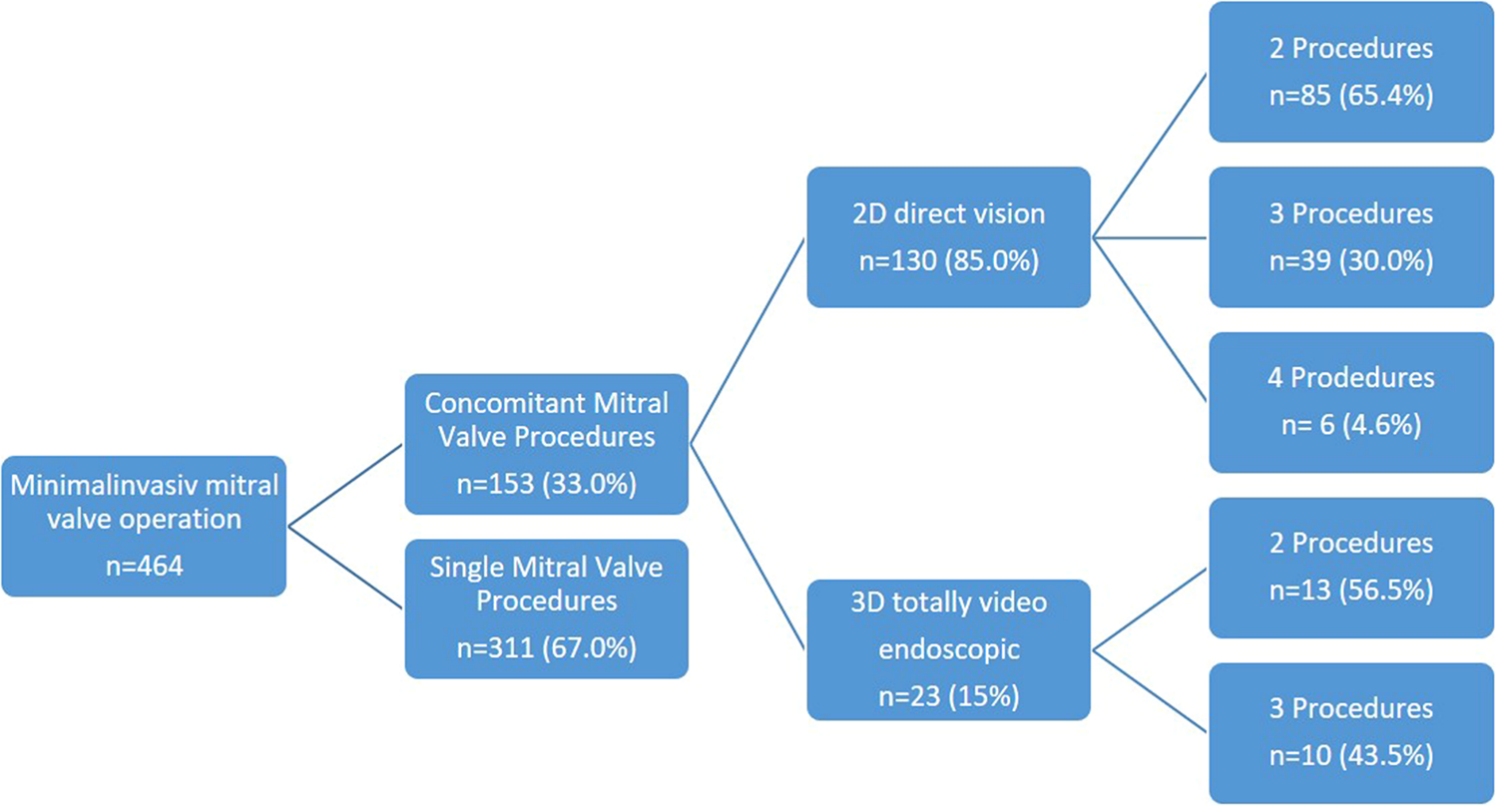
Minimal invasive procedures.

**Figure 2:**
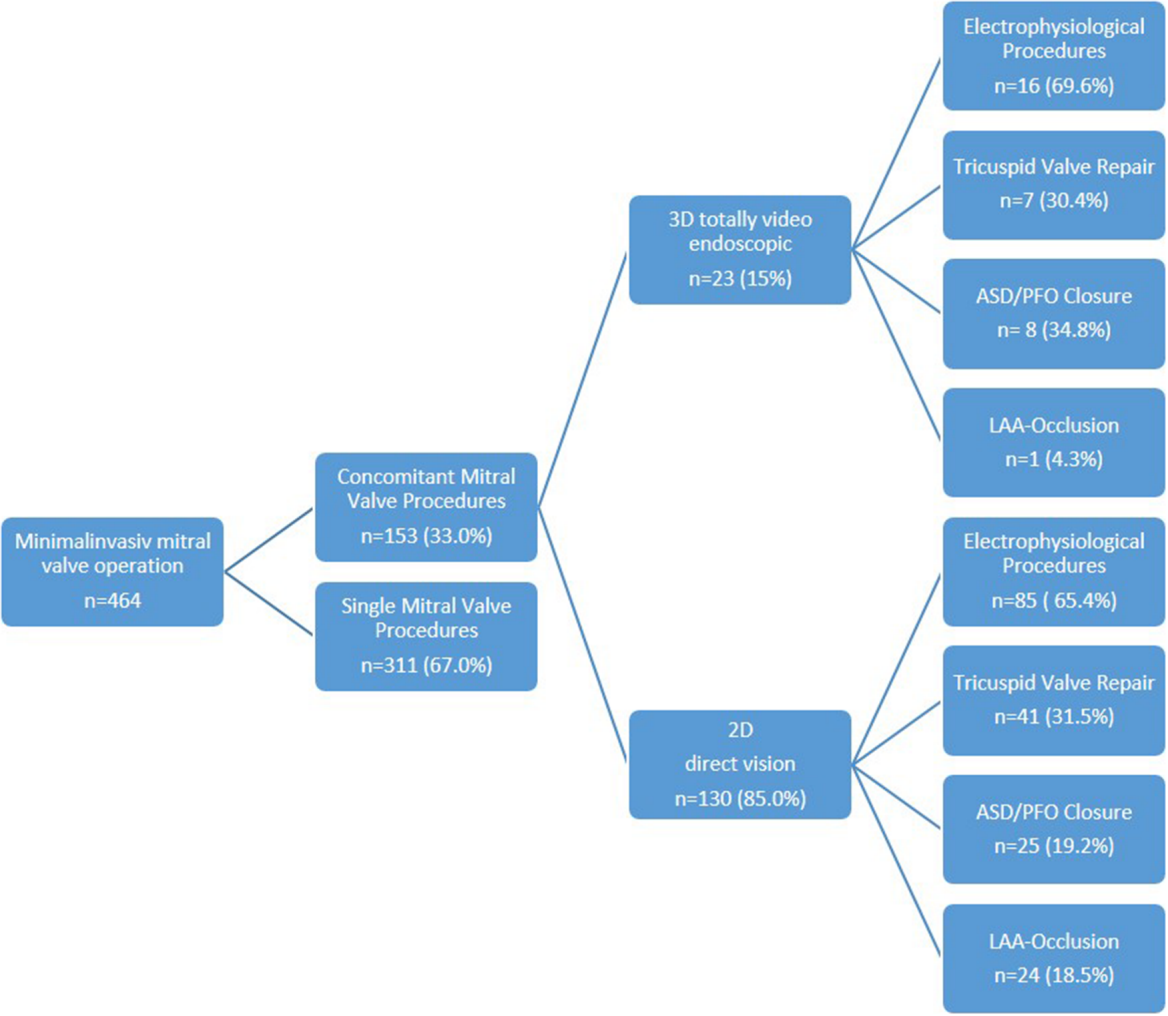
Minimal invasive mitral valve repair and concomitant procedures: 2D and 3D.

Overall, 98 patients (64.1%) underwent a combination of two procedures (2D *n* = 85, 3D *n* = 13), 49 patients (32.0%) a combination of three procedures (2D *n* = 39, 3D *n* = 10), and 6 patients (3.9%) a combination of four procedures (2D *n* = 6, 3D *n* = 0) (Figure 1). Among these patients, 52 presented with tricuspid valve insufficiency requiring surgical repair, while 34 patients had an associated atrial septal defect (ASD) or patent foramen ovale (PFO). Additionally, 101 patients presented with atrial fibrillation necessitating an electrophysiological procedure, with 25 patients undergoing left appendage closure (Figure 2 and Table 2).

**Table 2:** Concomitant procedures.

** **	*N* = 153
Electrophysiological procedure	101 (66.0%)
LAA-occlusion	25 (16.3%)
Tricuspid valve repair	52 (34.0%)
ASD-closure	11 (7.2%)
PFO-closure	23 (15.0%)

### Preoperative evaluations

All patients underwent a preoperative CT scan, a coronary angiogram, and a high-quality echocardiogram to assess the suitability of a minimally invasive approach.

### Operative setting

All minimally invasive mitral valve operations were performed under general anesthesia and with intraoperative transesophageal echo examination. Two different approaches were performed during the study, as detailed below.

### 2D direct vision approach

Throughout the study period, advancements in technology significantly influenced the progression of minimally invasive surgical approaches. Initially, mitral valve repair was conducted using a 2D video-assisted endoscopic method with a right-sided thoracotomy for direct visualization. Subsequently, between January 2009 and November 2016, procedures transitioned to utilizing a 6- to 8-cm skin incision in the submammary crease, coupled with a 1-cm port incision in the anterior axillary line to support 2D videoendoscopy. To optimize visualization of the atrioventricular valves and both atria, as well as to facilitate instrument manoeuvrability, a soft tissue retractor was inserted and a small rib spreader was utilized.

### 3D totally endoscopic approach

**Figure 3:**
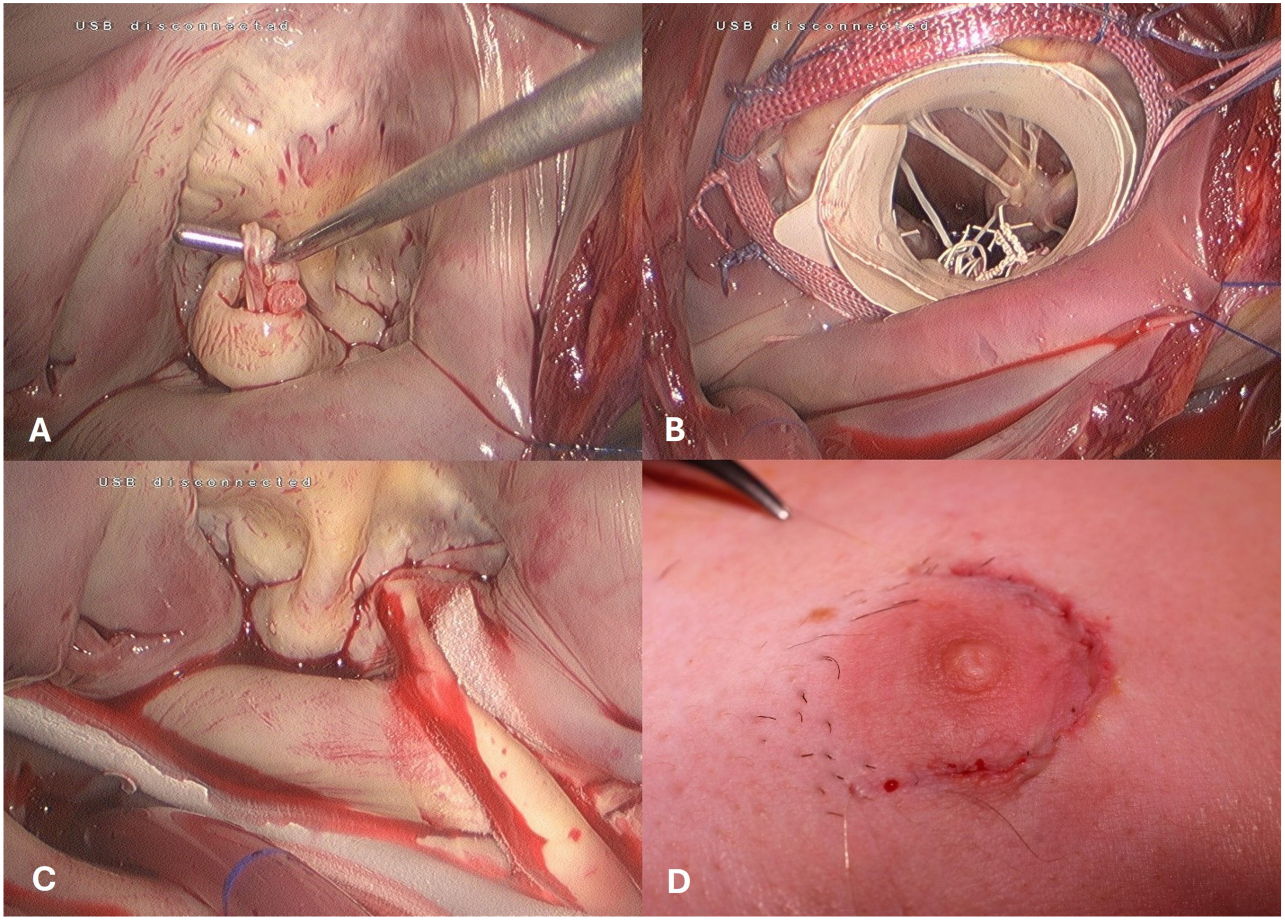
Intraoperative view of the totally endoscopic access. (**A**) Mitral Valve Prolaps. (**B**) Mitral valve repair using the paper roll. (**C**) concomitant cryo-maze procedure. (**D**) skin closure -periareolar inscision.

In December 2016, a transition was made to a fully endoscopic approach using a 3D endoscope (Aesculap Einstein Vision, Aesculap, Tuttlingen, Germany) for a group of 23 patients. In this approach, a 3.5- to 4.5-cm periareolar incision was made in male patients and a 4- to 5-cm incision in the submammary crease in female patients. This incision served as the working port after the insertion of a soft tissue retractor, eliminating the need for a rib spreader. This created an average working space of 3 × 1.5 cm through the thoracic wall. A 1-cm incision for the video port was made in the anterior axillary line for the insertion of the 3D endoscope (Figure 3A).

In both surgical approaches, cardiopulmonary bypass was initiated by arterial and venous cannulation through the right femoral vessels and the right jugular vein, respectively. The choice between percutaneous and surgical cut down for cannulation depended on the vessel size as measured in the preoperative CT scan.

For the percutaneous approach, Proglide (Abbot Vascular, Belgium) was utilized for arterial vessel closure. Alternatively, the surgical cut down involved making a 2.5-cm skin incision, through which the femoral artery was directly cannulated. Additionally, a leg perfusion cannula was placed when deemed necessary.

An antegrade cardioplegia line was then installed and passed through the working port. Cardiac arrest was induced using antegrade blood cardioplegia, administered at 20-minute intervals.

After opening the left atrium through the interatrial groove, the mitral valve operation was performed using standard surgical techniques such as artificial chordae, triangular resection, and sliding plasty. In cases of repair and difficult exposure of the subvalvular mitral valve apparatus, the previously described “paper roll” method was used (13). Adjunct procedures were then carried out as necessary (Figures 3B,C). Following bypass weaning, a single chest tube was inserted into the right pleural cavity, and an intercostal block with ropivacaine hydrochloride was established for pain relief. The skin was closed with an intracutaneous running suture (Figure 3D).

### Electrophysiological interventions

**Figure 4:**
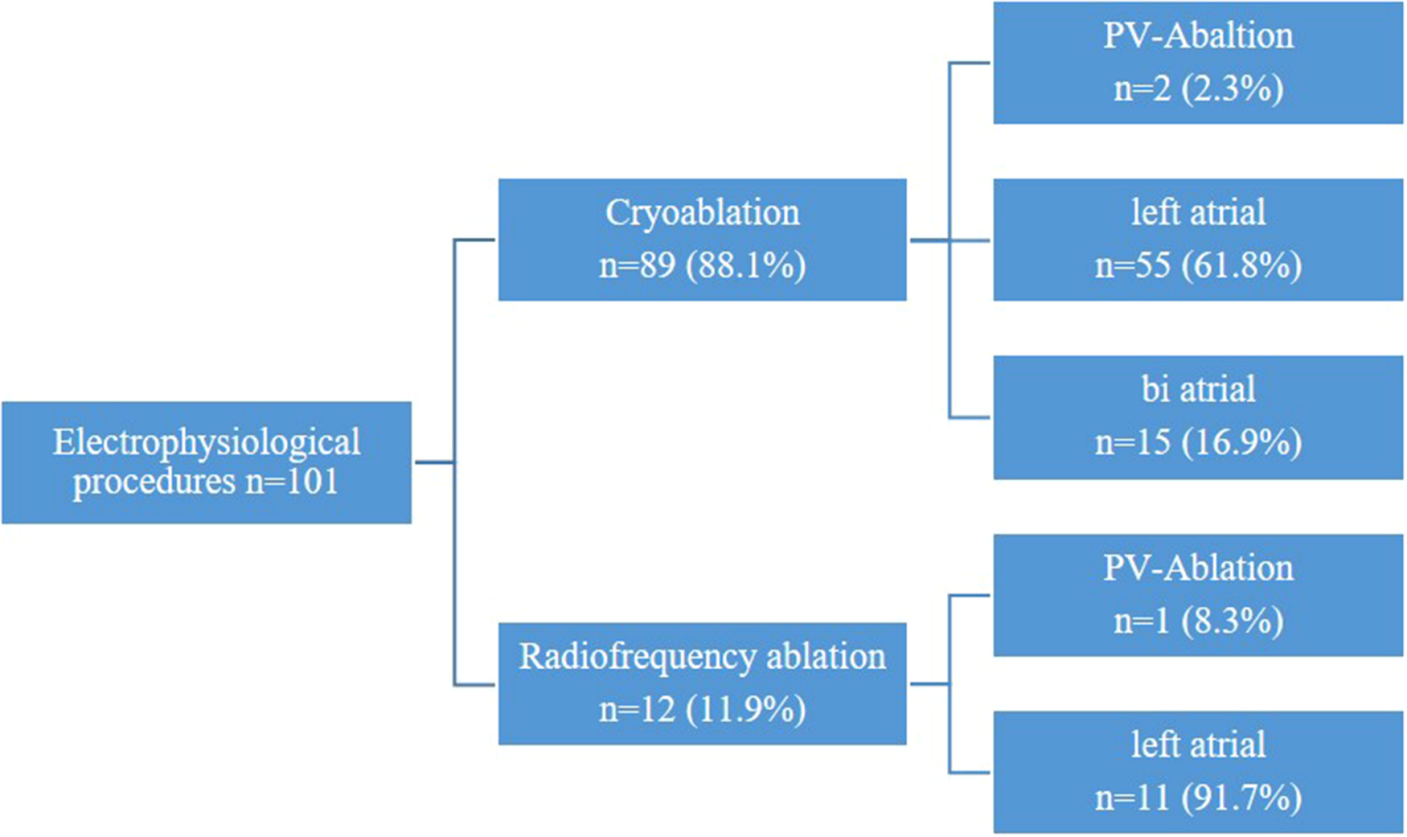
Electrophysiological procedures.

During the study period, two distinct ablation modalities were employed: unipolar radiofrequency ablation and cryoablation using CryoForm® (Articure Europe B.V., Amsterdam, Netherlands) (Figure 4). The duration of CryoMaze ablation was 120 s for the left atrium and 90 s for the right atrium.

Left atrial lesions were established utilizing a modified Cox III technique. Initially, the left atrial endocardial maze procedure was conducted, during which the pulmonary veins were encircled with the cryoprobe to create a box lesion. Subsequently, a linear lesion was drawn from the left lower pulmonary vein towards the posterior mitral valve annulus P3. Additionally, various approaches such as LAA amputation, overswung lesions, and circumferential lesions were implemented.

In cases requiring biatrial maze procedures, the right atrial lesions were performed subsequent to closure of the left atriotomy. The right atrial endocardial maze procedure was executed by extending the atrial incision line into both the superior and inferior vena cavae. A linear lesion was then delineated from the atrial incision at the free edge to the tricuspid valve annulus (posterior leaflet). Lastly, a line was drawn from the free edge of the right atrium across the fossa ovalis to the coronary sinus.

Concerning endocardial pulmonary vein ablation, the right pulmonary vein was encircled to form an island, followed by the isolation of the left pulmonary veins. The two islands were subsequently connected at the posterior wall of the left atrium.

### Mitral valve pathologies

The indication for surgery was mitral valve stenosis in 14 patients (9.2%), while the majority of patients (*n* = 139, 90.8%) presented with moderate (*n* = 4, 2.6%) to severe (*n* = 147, 96.1%) mitral valve insufficiency. A subset of patients exhibited specific pathologies, including leaflet prolapse in 100 patients (65.4%), a cleft in 4 patients (2.6%), commissural prolapse in 3 patients (2.0%), or a combination of previously described pathologies.

**Figure 5:**
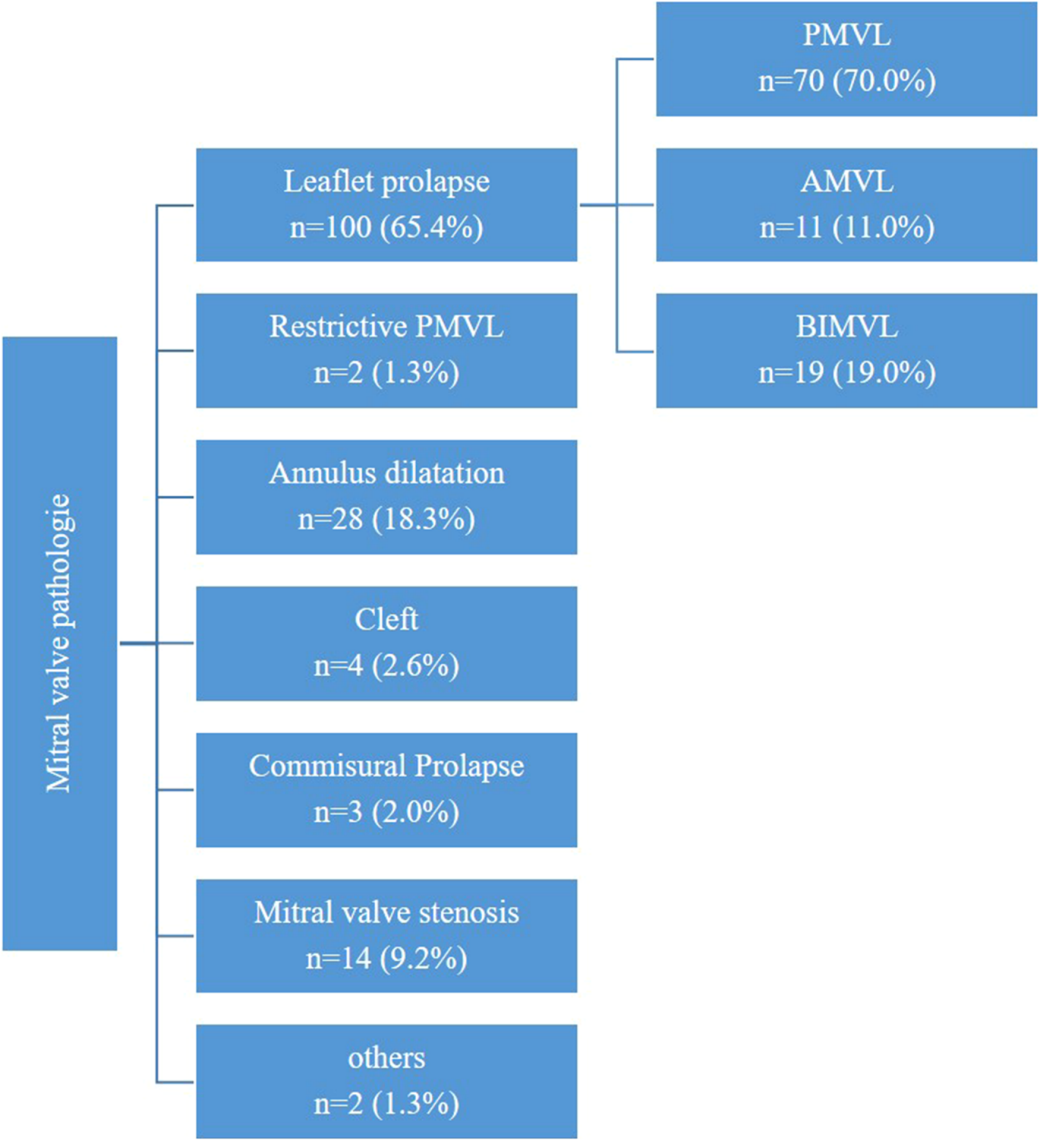
Mitral valve pathologies.

A detailed description of predominant mitral valve pathologies is provided in Figure 5.

### Data and statistics

The study followed the guidelines established in the Declaration of Helsinki (2013). Data collection was retrospective, and coding was implemented to ensure patient ID anonymization. Statistical analysis was performed using SPSS 24.0 (IBM SPSS, Chicago, IL). Continuous data were presented as mean ± standard deviation, while categorical variables were reported as absolute numbers and percentages. Patient survival and freedom from reoperation were assessed using the Kaplan-Meier method.

## Results

### Concomitant procedures

Among patients undergoing two concomitant procedures (*n* = 98), the most prevalent combination comprised mitral valve repair and the maze procedure, observed in 38 patients (38.8%). Mitral and tricuspid valve repair were conducted in 22 patients (22.5%), while PFO closure was performed in 15 patients (15.3%).

For patients undergoing three concomitant procedures (*n* = 49), a combination involving mitral valve repair, the maze procedure, and LAA occlusion was administered in 17 patients (34.7%). Furthermore, mitral and tricuspid valve repair along with the maze procedure were performed in 13 patients (26.5%).

Among patients undergoing four concomitant procedures (*n* = 6), two patients (33.3%) underwent a combination of mitral and tricuspid valve repair, the maze procedure, and LAA occlusion. A combination of mitral and tricuspid valve repair, the maze procedure, and ASD closure was performed in three patients. One patient underwent a biological mitral valve replacement, tricuspid valve repair, maze procedure and LAA occlusion.

LAA closure was considered an additional procedure, whether or not the MAZE procedure or PV ablation was performed, as it was not done for every patient undergoing an electrophysiological procedure. In patients who had the MAZE procedure but did not undergo LAA occlusion, the LAA was excised electrophysiologically using circumferential lesions around it.

A detailed breakdown of the distribution of concomitant procedures is provided in Table 3.

**Table 3:** Concomtiant procedures.

2 concomitant procedures	*N* = 98 (64.1%)
Mitral valve repair & MAZE	38 (38.8%)
Mitral valve repair & Tricuspid valve repair (TVR)	22 (22.5%)
Mitral valve repair & PFO Closure	15 (15.3%)
Mitral valve repair & ASD Closure	5 (5.1%)
Mitral valve repair & PV-Ablation	4 (4.1%)
Mitral valve repair & Myxoma-Resection	1 (1.0%)
Mitral valve replacement (bio) & TVR	3 (3.1%)
Mitral valve replacement (bio) & MAZE	2 (2.0%)
Mitral valve replacement (mech) & TVR	2 (2.0%)
Mitral valve replacement (mech) & PV-Ablation	1 (1.0%)
Mitral valve replacement (mech) & MAZE	3 (3.1%)
Others	2 (2.0%)
3 concomitant procedures	*N* = 49 (32.0%)
Mitral valve repair & MAZE & LAA-Occlusion	17 (34.7%)
Mitral valve repair & MAZE & LAA-Clip	3 (6.1%)
Mitral valve repair & TVR & MAZE	13 (26.5%)
Mitral valve repair & MAZE & PFO Closure	7 (14.3%)
Mitral valve repair & MAZE & ASD Closure	2 (4.1%)
Mitral valve repair & PV-Ablation & LAA-Occlusion	1 (2.0%)
Mitral valve repair & MAZE & Myxoma-Resection	1 (2.0%)
Mitral valve repair & TVR & PFO Closure	1 (2.0%)
Mitral valve repair & TVR & ASD Closure	1 (2.0%)
Mitral valve repair & TVR & PV-Ablation	1 (2.0%)
Mitral valve replacement (bio) & TVR & MAZE	1 (2.0%)
Mitral valve replacement (mech) & MAZE & LAA-occlusion	1 (2.0%)
4 concomitant procedures	*N* = 6 (3.9%)
Mitral valve repair & TVR & MAZE & LAA-Occlusion	2 (33.3%)
Mitral valve repair & TVR & MAZE & ASD	3 (50%)
Mitral valve replacement (bio) & TVR & MAZE & LAA-Occlusion	1 (16.7%)

### Mitral valve repair/replacement

**Figure 6:**
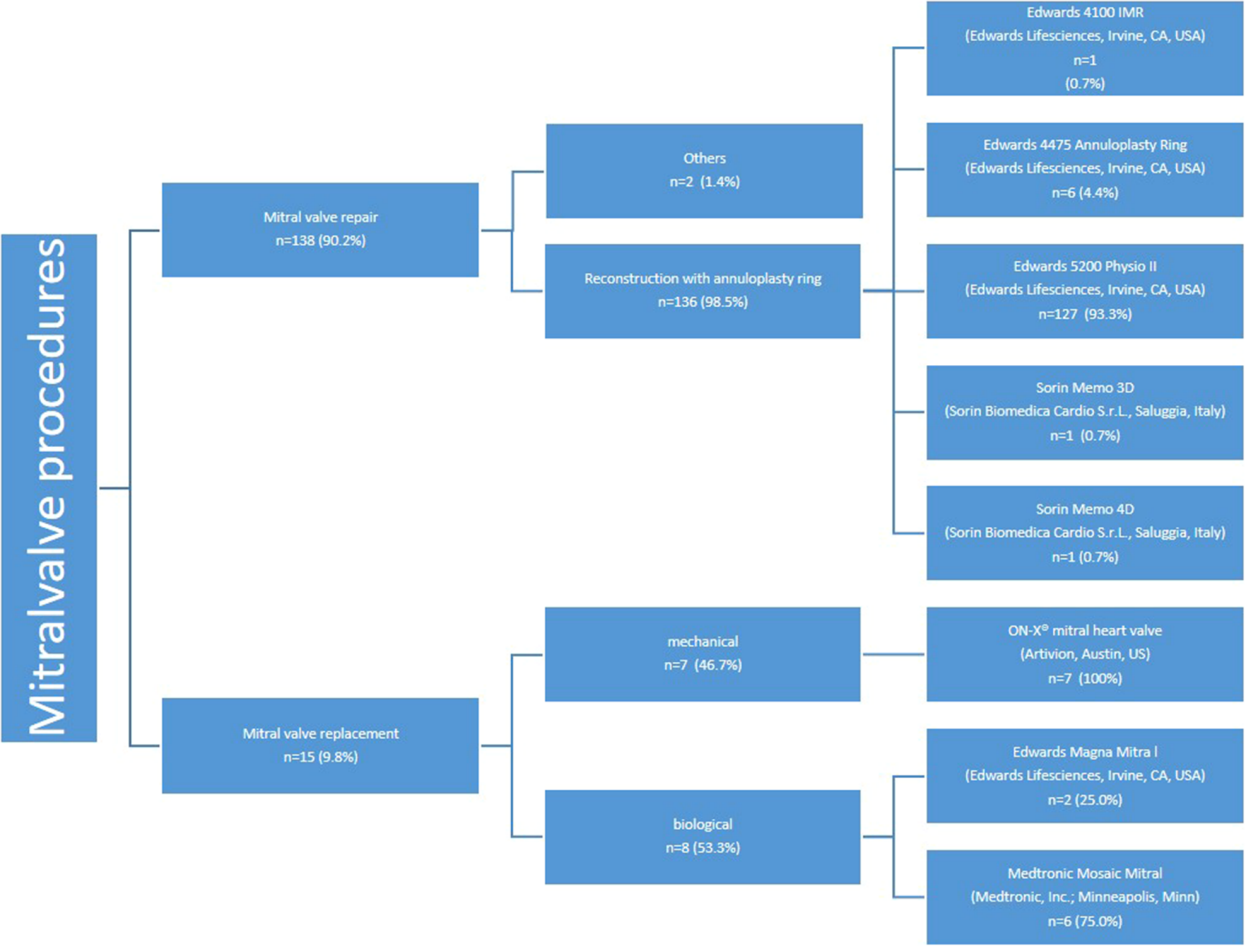
Mitral valve procedures: implants used for mitral valve replacement and repair.

Mitral valve repair was successfully completed in 136 patients. Additionally, 15 patients (9.8%) with underlying mitral valve stenosis underwent scheduled mitral valve replacement, with 7 receiving mechanical valves and 8 receiving biological valves (see Figure 6). The ON-X® mitral heart valve (Artivion, Austin, US) was utilized for all patients undergoing mechanical mitral valve replacement, while for biological mitral valve replacement, two patients (25.0%) received the Edwards Magna Mitral valve and six patients (75.0%) received the Medtronic Mosaic Mitral valve.

Additionally, one patient underwent closure of a paravalvular leak (PVL) associated with a previously implanted mitral valve prosthesis, while another patient received an Alfieri stitch due to severe annular calcification (Table 2).

Mitral valve repair procedures were chosen to address specific pathologies. Neochord implantation was used in 75 patients (49.0%), while other repair methods were utilized in 34 patients (15.7%). Furthermore, annuloplasty alone was performed in 37 patients (24.1%). Detailed descriptions of the repair techniques are provided in Table 4.

**Table 4:** Mitral valve procedures.

Mitral valve procedures	*N* = 153 (100%)
Replacement	15 (9.8%)
Annuloplasty only	37 (24.2%)
Non—loop repair	24 (15.7%)
Loop repair	75 (49.0%)
Others	2 (1.3%)
Repair techniques	*N* = 101 (66.0%)
Neochord-implantation (only)	52 (51.5%)
Resection (only)	13 (12.9%)
Cleftclosure (only)	5 (5.0%)
Secondary chordae transfer (only)	1 (1.0%)
Commissuroplasty	3 (3.0%)
Resection & neochords	2 (2.0%)
Neochords & cleft closure	11 (10.9%)
Resection & neochords & secondary chordae transfer	1 (1.0%)
Resection & neochords and sliding plasty	6 (5.9%)
Resection & secondary chordae transfer	2 (2.0%)
Neochords & secondary chordae transfer	1 (1.0%)
Neochords & cleft closure & myxomaresection	1 (1.0%)
PVL-closure	1 (1.0%)
Alferi-stich	1 (1.0%)

The most commonly used annuloplasty ring was the Edwards Physio II, utilized in 127 patients (93.3%) (Figure 6). The predominant sizes for the mitral annuloplasty ring were 34 mm in 32 patients (23.5%) and 36 mm in 28 patients (20.6%). The details of the other ring sizes are summarized in Table 5.

**Table 5:** Size of annuloplasty ring implanted in the mitral or tricuspid position.

Mitral valve annuloplasty ring size (mm)	*N* = 136
28	7 (5.2%)
30	20 (14.7%)
32	21 (15.4%)
34	32 (23.5%)
36	28 (20.6%)
38	14 (10.3%)
40	14 (10.3%)
Tricuspid valve annuloplasty ring size (mm)	*N* = 52
28	4 (7.7%)
30	13 (25.0%)
32	19 (36.5%)
34	9 (17.3%)
36	7 (13.5%)

### Tricuspid valve repair

In all patients who underwent tricuspid valve repair, the indication was a functional tricuspid valve insufficiency due to ring dilatation. Therefore, it was possible to perform a tricuspid valve repair in all such patients. The predominant size of the tricuspid annuloplasty ring was 32 mm (*n* = 19, 36.5%) or 30 mm (*n* = 13, 25.0%). Table 5 summarizes the implanted ring size.

### Perioperative and in-hospital outcome

No significant differences in postoperative or perioperative complications were observed between the 3D totally endoscopic and 2D direct vision approaches. Additionally, there were no significant differences in total operation time or aortic cross-clamp time between the two groups. However, CPB time was longer for the 2D group (250 ± 44 min) compared to the 3D group (221 ± 44 min).

The discharge echocardiogram showed a competent mitral valve in 152 patients (99.3%), with mild insufficiency observed in one patient (0.7%). The tricuspid valve was competent in 149 patients (97.4%), with mild insufficiency observed in one patient (0.7%) and moderate insufficiency in 3 patients (2.0%).

In the discharge ECG, successful conversion to sinus rhythm was observed post-operation in 86 (85.1%) of the 101 patients who underwent the electrophysiological procedure, while the remaining 15 patients (14.9%) were discharged with atrial fibrillation under medical therapy controlling heart rate and anticoagulation.

Postoperative data summarized in Table 6.

**Table 6:** Postoperative course.

Postoperative outcome	*N* = 153
ICU time (days) median	1
Operation time (min) median	325
Perfusion time (min) median	222
Aortic cross clamp time (min) median	142
ECMO Implantation	2 (1.3%)
Postoperative SAM resulting in reoperation	1 (0.7%)
Conversion to full sternotomy	1 (0.7%)
Acute peripheral ischaemia	3 (2.0%)
Acute kidney injury	3 (2.0%)
Intermittent dialysis	2 (1.3%)
Pulmonary embolism	1 (0.7%)
Prolonged neurological deficit	7 (4.6%)
Transient neurological deficit	3 (2.0%)
Pacemaker-Implantation	10 (6.5%)
Post MAZE-Procedure	3 (2.0%)
AV-Block Grade III	7 (4.6%)
Bleeding complication	10 (6.5%)
Lymphatic fistula	5 3.3%)
Sepsis	1 (0.7%)
30 day mortality	1 (0.7%)
Mitral valve insufficiency discharge	
Grade 0 or –I	152 (99.3%)
Grade 1	1 (0.7%)
Tricuspid valve insufficiency discharge	
Grade 0	149 (97.4%)
Grade I	1 (0.7%)
Grade II	3 (2.0%)

### Perioperative and in-hospital complications

Postoperative data summarized in Table 6.

One patient required conversion to sternotomy due to difficult to control bleeding during the operation.

Extracorporeal membrane oxygenation (ECMO) implantation was necessary due to pulmonary edema in two patients. The mean cardio-pulmonary bypass time was 183 min and the mean aortic cross clamp time 98 min in these two patients.

Another patient developed systolic anterior motion perioperatively, which was underestimated in the intraoperative transesophageal echocardiogram, resulting in reoperation on the first postoperative day. During this second procedure, the annuloplasty ring was upsized, and the repair was successful.

Ten patients experienced severe bleeding requiring revision for right-sided hemothorax; in these cases, it was possible to perform the surgical revision in a minimally invasive way.

Pacemaker implantation was necessary in 10 patients, three of whom additionally underwent a maze procedure. Surgical revisions of lymphatic fistulas were needed in five patients.

Seven patients presented a prolonged neurological deficit;

Four of these patients had a tonic-clonic seizure, one of whom had a history of stroke. Two patients who presented perioperative stroke also presented a tonic-clonic seizure.

Two patients developed transient peripheral nervous peroneal lesions postoperatively, while one experienced temporary disability in lifting the left arm (brachial plexus injury), attributed to surgical pressure injury.

Remarkably, one patient with hemiparesis, showing no signs of stroke on cerebral CT scans, fully recovered during hospitalization.

### Mortality

The 30-day mortality rate was 0.7%, with one patient dying from respiratory insufficiency.

**Figure 7:**
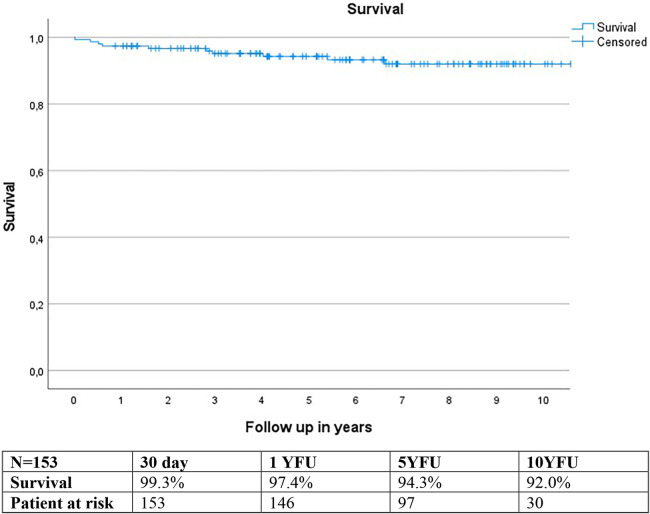
Kaplan meier survival curve.

Estimated survival using the Kaplan-Meier method was 97.4% at 1-year follow-up (YFU), 94.3% at 5YFU, and 92.0% at 10YFU (Figure 7).

Two patients died during the study period due to cardiac reasons, while nine others died from non-cardiac causes.

### Freedom from reoperation

**Figure 8:**
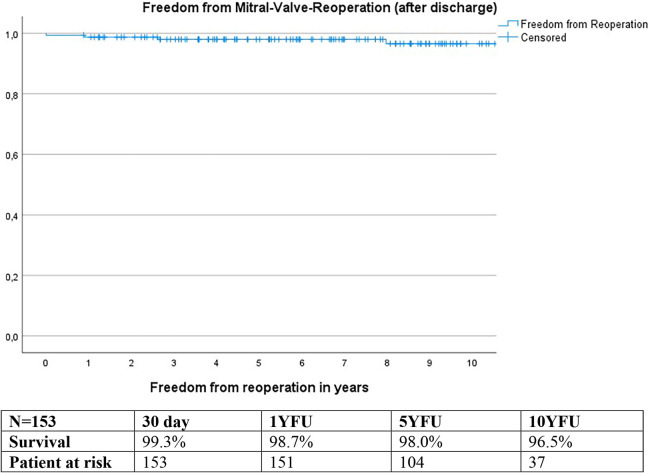
Freedom from mitral valve reoperation: kaplan meier curve.

Calculations using the Kaplan-Meier method revealed freedom from reoperation of 99.3% at 30 days, 98.7% at 1YFU, 98.0% at 5YFU, and 96.5% at 10YFU (Figure 8).

Reoperation was necessary in two patients due to valve degeneration, and one patient developed endocarditis. Out of these, one patient had a biological mitral valve replacement, while the other two underwent three or four concomitant procedure repairs.

## Discussion

Mitral valve repair stands as the “gold-standard” for managing patients with mitral valve insufficiency, boasting commendable long-term outcomes, particularly in organic MR. However, addressing intricate pathologies poses a challenge, even for experienced surgeons, given the complexity of the valve's constituent parts, including the mitral annulus, leaflets, chords, papillary muscles, and ventricle, all necessitating meticulous evaluation and treatment during surgery (22–26).

The emergence of minimally invasive surgical techniques in the mid-1990s marked a significant advancement in cardiac surgery. Studies affirm that minimally invasive mitral valve surgery, conducted via minithoracotomy, is as safe and effective as conventional open-heart procedures (9, 10, 12, 14, 27, 28). Patients benefit from enhanced well-being, cosmetic outcomes, and expedited return to normal activities compared to traditional surgery (1–5, 7, 11, 17).

Nevertheless, limitations in visualization have hindered further minimization of invasiveness. Conventional endoscopes offer only 2D images, limiting depth perception and complicating complex procedures. Consequently, direct vision remains paramount, especially in intricate cases where precise visualization is critical (1, 6, 11).

The introduction of 3D endoscopes represents a pivotal leap forward, enabling improved visualization and enhanced surgical capabilities. With 3D visualization, surgeons can perform intricate procedures entirely endoscopically, achieving outcomes akin to conventional minimally invasive techniques (6, 11, 17, 29). The adoption of the 3D approach at our institution in December 2016 further underscores the feasibility and efficacy of this technique.

In our study cohort, both the 2D direct vision approach and the 3D totally endoscopic approach were employed. Despite concerns regarding prolonged cardiopulmonary bypass and aortic cross-clamp durations, our results reveal an overall 30-day mortality rate of 0.7%, comparable to reported rates for single mitral valve repair procedures ranging from 0.2%–2.6% (4, 8, 10, 15, 16, 18, 27) and even superior to the rate reported for full sternotomy (3.0%) (9, 12, 15, 16, 27).

Moreover, we demonstrated high rates of successful repair, even in cases involving complex pathologies and concomitant procedures. While critics may question the feasibility of implementing challenging repair techniques in complex cases, our findings suggest otherwise. We achieved a remarkable success rate (99.7%) of repair, even in cases involving anterior or bileaflet prolapse or commissural prolapse, which is comparable with to literature for single mitral valve repair ranging from 94 to 99.4% (4, 5, 8). Additionally, all additional procedures were successfully performed, with a conversion rate of 0.7% due to severe bleeding, which is slightly lower than reported rates in the literature (2%–5%) (2, 4, 5, 8, 16).

For patients undergoing mitral valve replacement, preoperative assessment indicated that repair was impossible due to the severity of mitral valve stenosis.

The study revealed that 4.6% of patients experienced prolonged neurological deficits, regardless of the number of concomitant procedures they underwent. The stroke rate is situated at the upper level compared to the rate described in the literature, following single mitral valve repair, which typically ranges from 0.7% to 5.0% (4, 5, 7, 30). Surprisingly, the majority of severe deficits occurred in patients who only underwent two procedures, surpassing the reported incidence of strokes following single mitral valve repair. Among the observed neurological events, three patients experienced grand mal seizures despite no evidence of stroke on imaging, likely due to prolonged cardio-pulmonary bypass time and hypotension. These findings underscore the importance of meticulous risk assessment and preoperative patient evaluation, which includes preoperative CT measurements focusing on technical feasibility. Effective management strategies are crucial to mitigate adverse neurological outcomes in cardiac surgery patients undergoing multi-procedure interventions.

The maze procedure for atrial fibrillation demonstrated an early success rate of 85.1% (patients discharged in sinus rhythm). Additionally, only three patients necessitated pacemaker implantation following the procedure. These outcomes not only meet but potentially exceed those reported in existing literature (19, 20).

While combined totally endoscopic mitral procedures offer numerous benefits, including reduced surgical trauma and enhanced cosmetics, longer extracorporeal bypass and aortic cross-clamp times may be considered drawbacks.

The complexity of the procedures, along with a higher incidence of additional interventions such as tricuspid valve repair (34.6%), ASD/PFO closure (34%), and the Maze procedure (66%), surpasses that documented in previous studies (4, 8, 17, 19, 21). Comparative rates ranged from 2.4% to 14.6% for tricuspid valve repair, 3.2% for ASD/PFO closure, and 9.5% to 100% for the Maze procedure. Consequently, the median perfusion duration (222 min) and aortic cross-clamp duration (142 min) in our cohort slightly exceeded the reported ranges in the literature, which typically span from 120 to 183 min for perfusion time and 88–122 min for cardiopulmonary bypass time (4, 8, 17, 19, 21). Most of the procedures were carried out by two experienced surgeons. However, a percentage of the surgeries were conducted by less experienced surgeons. This might result in longer bypass and cross-clamp times. No significant differences in postoperative or perioperative complications were noted between the 3D totally endoscopic and 2D direct vision approaches. Furthermore, there were no notable variations in total operation time or aortic cross-clamp time between the two groups. However, the cardiopulmonary bypass (CPB) time was significantly longer in the 2D group. This discrepancy may be attributed to changes in operative strategy and the increasing expertise of the surgeons over time. The prolonged extracorporeal bypass and aortic cross-clamp times led to lung edema and subsequent ECMO implantation in two patients, representing only 1.3% of the total patient cohort.

Furthermore, longer perfusion times may compromise leg perfusion, particularly in patients with small groin vessels. In our cohort, three patients experienced compartment syndrome of the lower leg, prompting the implementation of routine antegrade perfusion cannula insertion into the femoral artery to mitigate this risk.

Freedom from reoperation rates at 1 and 5 years postoperatively were 98.7% and 98.0%, respectively, comparable to those reported for single mitral valve procedures in previous studies 93.3–97.1% at 1YFU and 93.3% to 96.6% at 5YFU (18, 29) and 92.9% at 10 YFU (18).

The estimated postoperative survival was 97.4% at one year, 94.3% at 5 years, and 92.0% at 10 years. Our data exhibit comparable, if not slightly superior, outcomes compared to the literature, where the range is 87.0%–93.4% at 5 years and around 74%–84.5% at 10 years (4, 18).

The majority of patients (63.4%) underwent two concomitant procedures, while 34.0% underwent three procedures. Nevertheless, our study demonstrated excellent outcomes, affirming the feasibility of minimal invasiveness even in complex mitral valve procedures requiring additional interventions. This approach, whether under direct vision or entirely through video endoscopy, upholds the quality of mitral valve repair without increasing complications compared to minimally invasive single mitral valve repair. Minimally invasive surgery is no longer limited to isolated mitral valve procedures but can be extended to selected patients with more complex mitral insufficiencies in conjunction with other pathologies.

## Conclusion

Our study demonstrates the feasibility and effectiveness of minimally invasive mitral valve surgery, even in complex cases with concomitant procedures. Continued advancements in surgical techniques and technology, along with careful patient selection and surgical expertise, will further optimize outcomes and expand the applicability of minimally invasive approaches in the treatment of mitral valve disease.

### Limitations

Our study, while insightful, is limited by its retrospective design and single-centre scope. Retrospective studies are prone to biases and incomplete data, impacting generalizability. Future research should prioritize well-designed, multicenter studies with longer follow-up to confirm and expand upon our findings, ensuring more comprehensive insights for clinical decision-making.

## Data Availability

The original contributions presented in the study are included in the article/Supplementary Material, further inquiries can be directed to the corresponding author.
